# Neutralizing antibody VRC01 failed to select for HIV-1 mutations upon viral rebound

**DOI:** 10.1172/JCI134395

**Published:** 2020-05-18

**Authors:** Evan M. Cale, Hongjun Bai, Meera Bose, Michael A. Messina, Donn J. Colby, Eric Sanders-Buell, Bethany Dearlove, Yifan Li, Emily Engeman, Daniel Silas, Anne Marie O’Sullivan, Brendan Mann, Suteeraporn Pinyakorn, Jintana Intasan, Khunthalee Benjapornpong, Carlo Sacdalan, Eugène Kroon, Nittaya Phanuphak, Robert Gramzinski, Sandhya Vasan, Merlin L. Robb, Nelson L. Michael, Rebecca M. Lynch, Robert T. Bailer, Amélie Pagliuzza, Nicolas Chomont, Amarendra Pegu, Nicole A. Doria-Rose, Lydie Trautmann, Trevor A. Crowell, John R. Mascola, Jintanat Ananworanich, Sodsai Tovanabutra, Morgane Rolland, on behalf of the RV397 Study Group

**Affiliations:** 1 Vaccine Research Center, National Institute of Allergy and Infectious Diseases, NIH, Bethesda, Maryland, USA.; 2 US Military HIV Research Program, Walter Reed Army Institute of Research, Silver Spring, Maryland, USA.; 3 Henry M. Jackson Foundation for the Advancement of Military Medicine, Inc., Bethesda, Maryland, USA.; 4 SEARCH, Thai Red Cross Research Center, Bangkok, Thailand.; 5 George Washington University, Washington, DC, USA.; 6 CHUM, Université de Montréal, Quebec, Canada.; 7 Department of Global Health, University of Amsterdam, Amsterdam, Netherlands.

**Keywords:** AIDS/HIV, Adaptive immunity, Bioinformatics

## Abstract

Infusion of the broadly neutralizing antibody VRC01 has been evaluated in individuals chronically infected with HIV-1. Here, we studied how VRC01 infusions affected viral rebound after cessation of antiretroviral therapy (ART) in 18 acutely treated and durably suppressed individuals. Viral rebound occurred in all individuals, yet VRC01 infusions modestly delayed rebound and participants who showed a faster decay of VRC01 in serum rebounded more rapidly. Participants with strains most sensitive to VRC01 or with VRC01 epitope motifs similar to known VRC01-susceptible strains rebounded later. Upon rebound, HIV-1 sequences were indistinguishable from those sampled at diagnosis. Across the cohort, participant-derived Env showed different sensitivity to VRC01 neutralization (including 2 resistant viruses), yet neutralization sensitivity was similar at diagnosis and after rebound, indicating the lack of selection for VRC01 resistance during treatment interruption. Our results showed that viremia rebounded despite the absence of HIV-1 adaptation to VRC01 and an average VRC01 trough of 221 μg/mL. Although VRC01 levels were insufficient to prevent a resurgent infection, knowledge that they did not mediate Env mutations in acute-like viruses is relevant for antibody-based strategies in acute infection.

## Graphical abstract

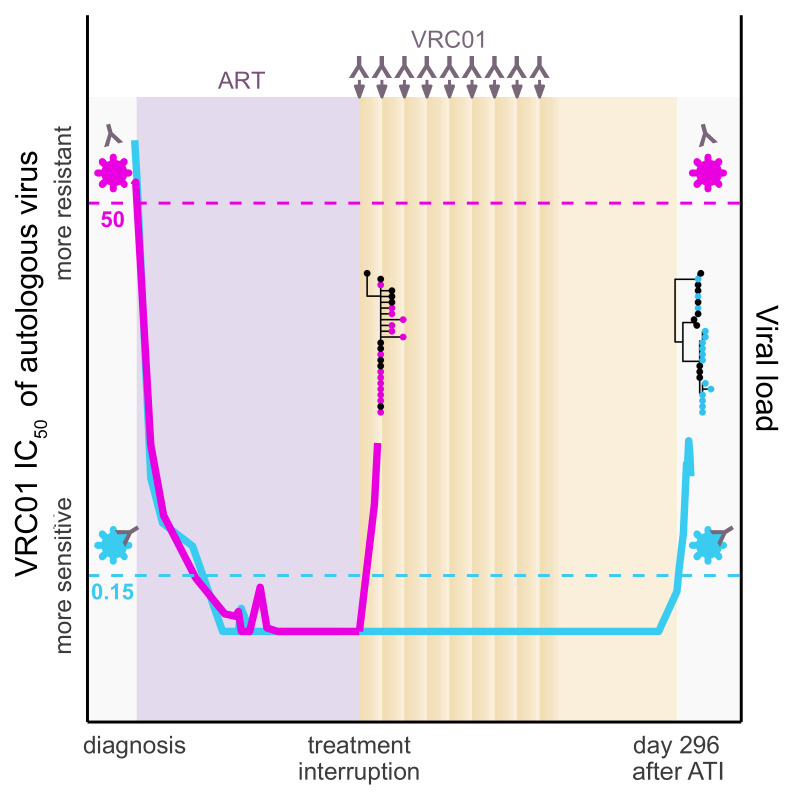

## Introduction

Analytic treatment interruption (ATI) studies can help evaluate strategies to mediate long-term remission in HIV-1–infected persons. An ATI study tested the impact of the administration of the broadly neutralizing antibody VRC01 (1). Eighteen participants who initiated ART in Fiebig I to III and were treated for 3 years (range: 2.3–6.6) were randomized to receive VRC01 (*n* = 13, 40 mg/kg) or placebo (*n* = 5) infusions, started the day ART was stopped and given every 3 weeks for 24 weeks or until ART was reinitiated. ART reinitiation occurred when a participant had confirmed viral load (VL) over 1,000 copies/mL. All participants rebounded (RNA ≥ 20 copies/mL) between 9 and 296 days after ART interruption. The time to rebound tended to be longer in the VRC01 group (median = 29 days) than in the placebo group (median = 14 days) (*P* = 0.05) (Supplemental Figure 1 and ref. 1; supplemental material available online with this article; https://doi.org/10.1172/JCI134395DS1). Except for the participant with suppressed viremia for 296 days, rebound occurred despite VRC01 serum concentrations above the target trough level of 50 μg/mL (Supplemental Figure 2) — a threshold informed by its half-maximal inhibitory concentration (IC_50_, estimated as < 50 μg/mL against 80% to 90% of circulating viruses) and its terminal half-life (14 ± 2.9 days) (2).

Prior clinical trials tested VRC01 infusions in chronically infected individuals (3, 4). They showed a temporary reduction in viremia and an increase in resistance to VRC01 neutralization. Because these participants had been infected for several years, their HIV-1 population was heterogeneous, as HIV-1 diversifies by approximately 1%/year in the *env* gene (5). In contrast, our participants were diagnosed at Fiebig I–III and their viral population should be homogeneous (6). Because participants started ART immediately after diagnosis, the viral diversity and reservoir should be limited upon ART cessation (7).

Because time to rebound varied across participants (Supplemental Figures 1 and 2), we investigated potential determinants of HIV-1 rebound. We sequenced HIV-1 at diagnosis and upon rebound. Participant-derived Env clones were tested for VRC01 neutralization sensitivity. We found no evidence that VRC01 selected for Env mutations or increased neutralization resistance during the short duration of replication. The temporary and modest delay in viral rebound in VRC01 recipients suggests that VRC01 concentrations were insufficient to afford sustained viral control.

## Results and Discussion

### No evidence of VRC01-mediated selection of mutations upon viral rebound.

**Figure 1:**
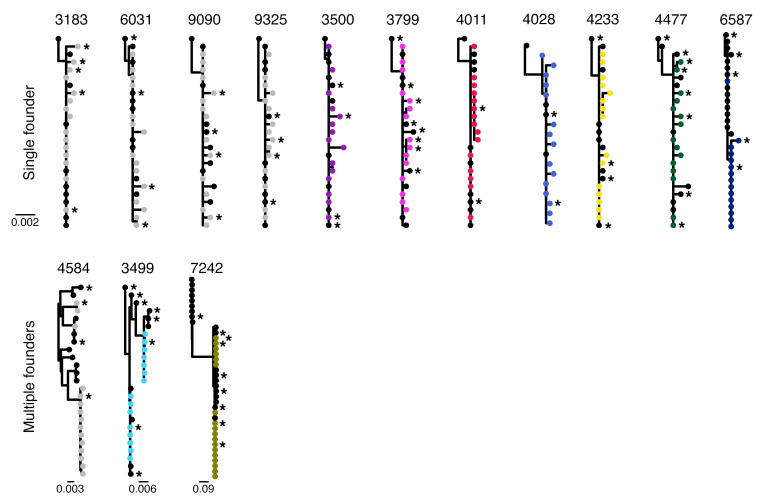
HIV-1 *env* sequences sampled at HIV-1 diagnosis and upon viral rebound were intermingled in phylogenetic trees. Phylogenies were reconstructed for 14 participants based on *env* sequences obtained from plasma samples collected in acute HIV-1 infection (pre-ART, shown in black) and upon viral rebound (following simultaneous VRC01 infusion and treatment interruption, after an average of 3 years on ART, shown in gray [placebo] or colors [VRC01]). Asterisks denote sequences tested with in vitro neutralization assays. The horizontal bar represents the number of substitutions per site. Four participants had *env* sequences corresponding to subtype B (3183, 4011) or a CRF01_AE–containing recombinant (3499, 7242).

**Figure 2:**
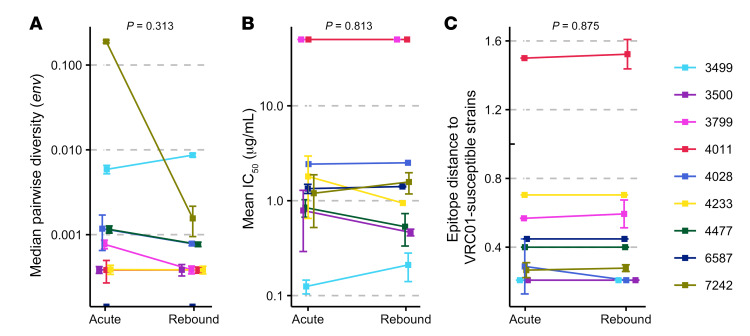
Sequence diversity, epitope distance, and VRC01 neutralization sensitivity values were similar in acute infection and upon viral rebound. Nine participants who received VRC01 infusions showed no significant difference over time for the median pairwise diversity across *env* sequences (median of 10 sequences in acute infection and 15 sequences upon rebound) (**A**), VRC01-specific IC_50_ values (**B**), and VRC01 epitope distances (**C**). Medians and standard deviations are represented and Wilcoxon’s signed-rank tests were performed.

To characterize the viruses that broke through VRC01 therapy, we sequenced 10 HIV-1 genomes at diagnosis (*n* = 18 participants) and 15 to 18 *pol* and *env* sequences upon rebound (*n* = 14) (Supplemental Table 1). Within-host phylogenetic trees showed that sequences across time points were intermingled (Figure 1 and Supplemental Figure 3), with some identical sequences at diagnosis and rebound. The median *env* pairwise diversity did not differ across time points (*P* = 0.31) (Figure 2A and Supplemental Figure 4a). We derived a consensus sequence from the sequences at diagnosis to represent the founder virus for each participant. For participants with single founders, sequences sampled upon viral rebound had on average 1 or 2 nucleotide mutations from the founder: 0.96 and 1.52 mutation in *pol* and *env*, respectively (Supplemental Figure 4, a and b). Hence, 73% (range 53–93) of *pol* and 60% (range 33–93) of *env* rebound sequences were at most one mutation away from the founder (Supplemental Figure 4c).

Furthermore, the few mutations observed upon rebound corresponded to what would be expected if HIV-1 replication had proceeded only after ART cessation and was lower than that expected if replication had proceeded during 2 to 6 years of treatment (Supplemental Figure 4A).

### Differences in time to rebound associated with VRC01 decay rates and neutralization sensitivity.

**Figure 3:**
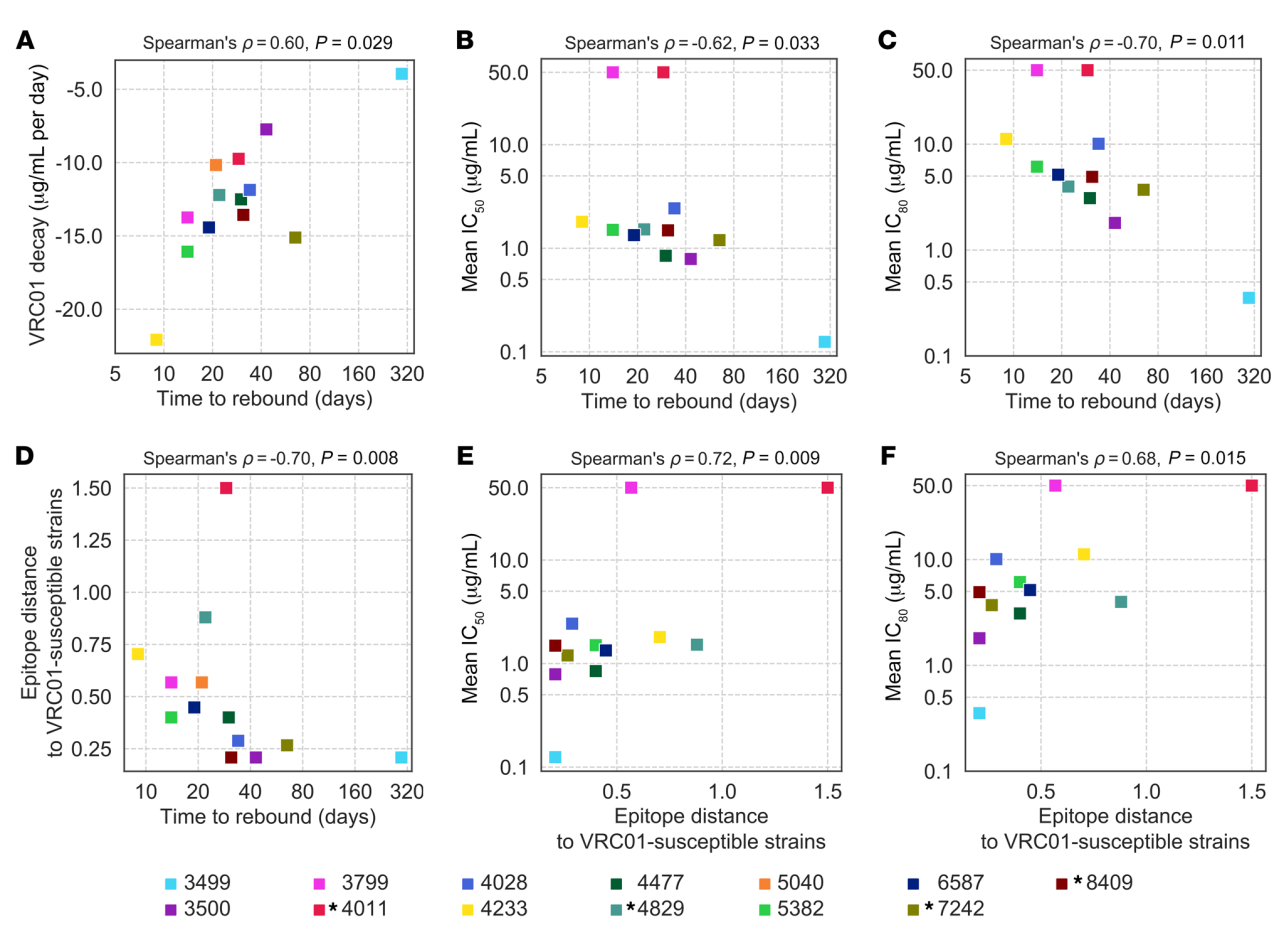
Relationship between VRC01 decay rate, neutralization sensitivity, and time to rebound in participants who received VRC01 infusions. Time to viral rebound was associated with the decay rate of VRC01 in serum after the first infusion (based on measurements on days 7, 14, and 21) (**A**). Time to viral rebound was also associated with the sensitivity to VRC01 using IC_50_/IC_80_ values corresponding to sequences sampled in acute infection (**B** and **C**) and to the VRC01 epitope distances (predicted from each participant’s sequences) (**E** and **F**). Predicted epitope distances were also correlated with the time to viral rebound (**D**). The time to rebound is the number of days between treatment cessation and an HIV-1 RNA test of 20 or more copies/mL. All 13 participants who received VRC01 infusions are represented; an asterisk before the participant ID indicates non-CRF01_AE infections.

All but one RV397 participant experienced viral rebound despite VRC01 serum concentrations above estimated IC_50_/IC_80_ values (Supplemental Figure 2). Participant 3499, who suppressed viremia for 296 days, had a VRC01 concentration of 12.5 μg/mL at rebound 20 weeks after his last infusion. For the other 12 participants, at rebound the average VRC01 concentration was 306 μg/mL (range 174–503) and the average trough before rebound was 221 μg/mL (range 144–319). Using VRC01 concentrations measured 7, 14, and 21 days after the first infusion, we calculated the decay rate of VRC01 in serum; a faster decay was associated with a more rapid viral rebound, *ρ* = 0.60, *P* = 0.03 (Figure 3A) (with decay rates calculated after the last infusion: *ρ* = 0.59, *P* = 0.04). Interestingly, participant 3499 showed the slowest decay rates after both the first (–1.80 μg/mL/day) and last (–3.95 μg/mL/day) infusion (median –13.27 μg/mL/day, range –7.7 to 22.1 for other participants).

To evaluate the neutralization sensitivity of viruses, we selected on average 5 *env* sequences per participant and generated pseudoviruses; there was no evidence that VRC01 sensitivity decreased between diagnosis and rebound (*P* = 0.81) (Figure 2B, with similar patterns for placebo recipients, Supplemental Figure 5). Two participants were infected by viruses that were already VRC01 resistant (IC_50_/IC_80_ > 50 μg/mL) and rebounded 14 (3799) and 29 (4011) days after ATI (Figure 2B). In contrast, participant 3499, who rebounded on day 296, presented the most VRC01-sensitive virus. Using IC_50_ values based on sequences sampled in acute infection (*n* = 12 participants), increased sensitivity to VRC01 was associated with a longer time to rebound: *ρ* = –0.62, *P* = 0.03 (Figure 3B) (IC_80_ values: *ρ* = –0.70, *P* = 0.01; Figure 3C). We also analyzed Hill coefficients, i.e., the slopes of the dose-response (VRC01 neutralization) curves for each participant; when compared with IC_50_/IC_80_ values, Hill coefficients are better indicators of the inhibition potency of an antibody at clinically relevant levels (8). The highest Hill coefficient (*H* = 1.48) was found for the participant who suppressed viremia for the longest time. Moreover, there was a significant positive relationship between Hill coefficients and time to rebound (*ρ* = 0.76, *P* = 0.01), illustrating that higher predicted values of therapeutic effectiveness were associated with longer suppression (Supplemental Figure 6). Finally, we noted that, after excluding participant 3499, a larger reservoir size before ATI (measured as the number of cells with HIV-1 DNA per million CD4^+^ cells) was associated with a faster time to rebound (*ρ* = –0.71, *P* = 0.010) (Supplemental Figure 7). Together, these results are evidence of VRC01 potency, albeit with therapeutically insufficient inhibition levels.

### Sequences with D at position 279 associated with earlier viral rebound.

**Figure 4:**
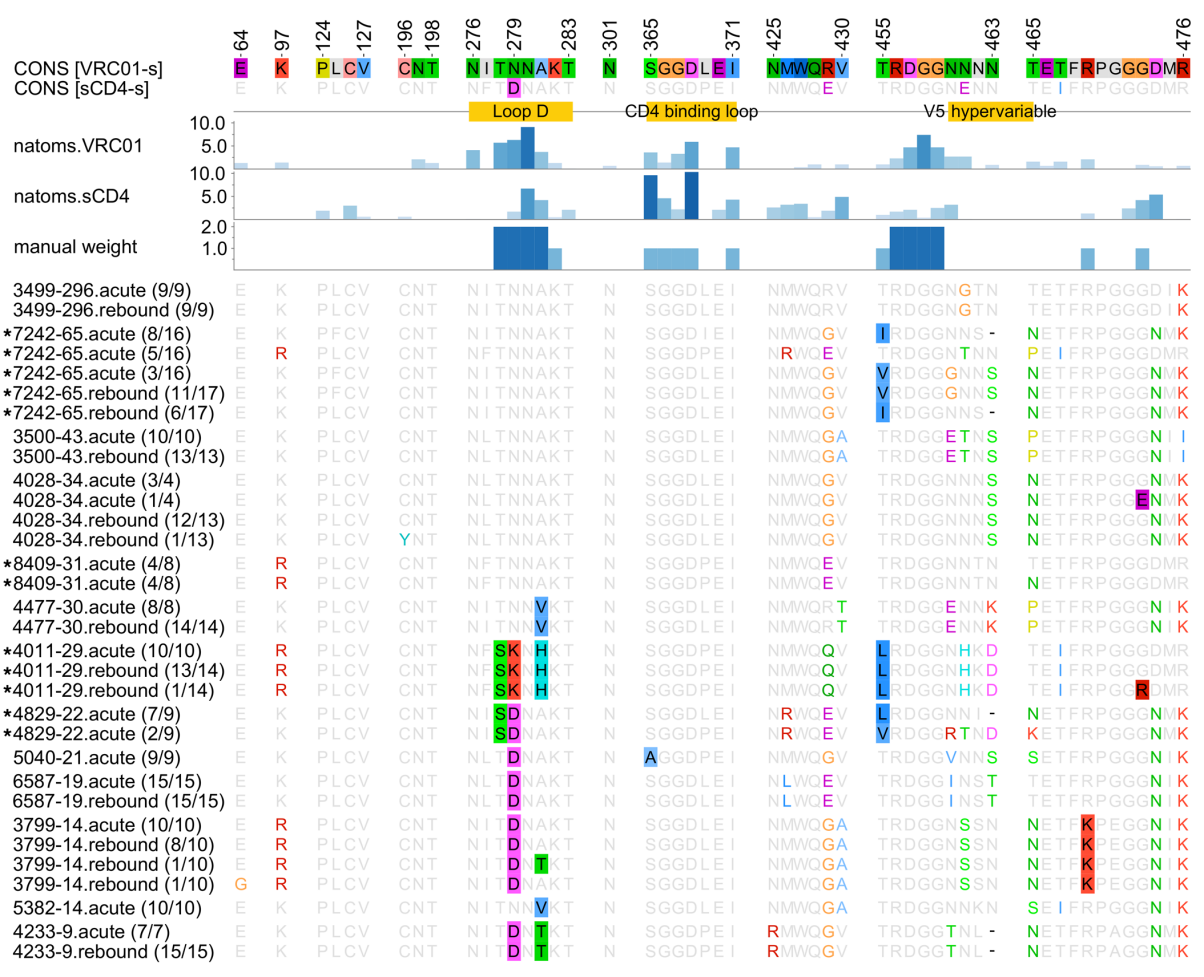
Comparison of sequences from RV397 participants to sequences known to be most sensitive to VRC01 or CD4. The top sequences correspond to the consensus residues found in the 5 sequences that were experimentally identified as most sensitive to VRC01 (VRC01-s) (9) and soluble CD4 (sCD4-s) (23). The importance of specific residues in the interaction with Env (measured by the number of VRC01 or sCD4 atoms that contact a residue) is indicated with darker colors for more influential residues. Core and rim epitope sites had a weight of 2 and 1, respectively. Sequences from the 13 participants who received VRC01 infusions are shown and labeled with a suffix corresponding to the time to rebound. Numbers in parentheses correspond to the number of sequences with the given epitope motif over the total sequences from a participant. Participants are ordered from longest to shortest time to rebound (from 296 to 9 days). Asterisks indicate participants with non-CRF01_AE infections.

To better characterize the genetic determinants of VRC01 responsiveness, we compared sequences from RV397 participants to sequences known to be sensitive to VRC01. We selected as references the 5 sequences that were the most sensitive among 136 strains tested experimentally (9). The VRC01 epitope motif was defined based on VRC01-Env complex structures (10–12). For participant 3499, who controlled viremia for 296 days, the epitope was almost identical to the consensus epitope of the 5 VRC01-sensitive references. In contrast, among participants who rebounded early, position 279 was enriched for D (consensus = N) (Figure 4); such a pattern was not seen among participants who did not receive VRC01 infusions (ref. 13 and Supplemental Figure 8). Viruses with 279D were significantly more resistant to VRC01 (*P* = 0.016) (Supplemental Figure 9), suggesting a role for 279D in the resistance to VRC01. However, sequences from prior studies testing VRC01 infusions presented equal proportions of N and D, with no association with VRC01 neutralization sensitivity (3, 4); likewise, there was no sensitivity difference among viruses with N (*n* = 90) or D (*n* = 43) in the 136 strains described above (*P* = 0.25) (9). Because 279D is frequently found across circulating sequences (34% of CRF01_AE sequences, Supplemental Table 2), it may provide an independent selective advantage. A previous study showed that 279D was associated with a reduced dependence on CD4 (14) and the inspection of Env-CD4 complex structures showed that it could be favored by CD4 (Supplemental Figure 10). Hence, the presence of 279D in half of subtype B and C sequences warrants further study.

### VRC01 epitope distance to VRC01-sensitive strains associated with delayed viral rebound.

To investigate whether sequence features could predict VRC01 sensitivity or time to rebound, we developed an epitope distance measure that integrates the interactions between Env and VRC01. This measure is derived from methods quantifying the relationship between the conservation of key epitope sites and neutralization breadth (15).

We compared summary epitope distances for RV397 sequences to those for the 5 most-VRC01-sensitive sequences. Participants with epitopes most similar to the VRC01-sensitive sequences showed the longest delay to rebound: *ρ* = –0.70, *P* = 0.01 (Figure 3D). In contrast, we found no relationship for 13 participants with no infusion (*ρ* = –0.23, *P* = 0.45) (Supplemental Figure 11). In silico VRC01 epitope distances were similar at diagnosis or rebound, further emphasizing that HIV-1 did not adapt to VRC01 (Figure 2C). Finally, we observed a positive relationship between our VRC01 epitope distance and the experimentally derived IC_50_/IC_80_ values: IC_50_ *ρ* = 0.72, *P* = 0.01; IC_80_ *ρ* = 0.68, *P* = 0.02 (Figure 3, E and F). If we include VRC01 and placebo participants, the significance of the relationship between epitope distance and neutralization sensitivity increased: IC_50_ values, *ρ* = 0.71, *P* = 0.002 (Supplemental Figure 12).

In summary, the analysis of viral sequences and Env VRC01 neutralization among 13 VRC01-treated participants provided 4 main conclusions regarding the impact of VRC01 infusion on the homogeneous HIV-1 populations typical of acute infection. First, sequences and neutralization values did not differ between diagnosis and rebound (*P* > 0.81). Hence, the impact of VRC01 was linked to whether, years before VRC01 infusion, the HIV-1 strain that infected each participant was sensitive to VRC01. The lack of VRC01-selected mutations upon rebound is most likely due to the near-absence of diversity among these participants’ sequences combined with the short duration of viral replication; these conditions are not conducive to the advent of variation that forms the basis of selection. Across all Env sequences from a participant with a single HIV-1 founder, on average 21 sites showed a mutation and only one of these sites (range 0–3) presented a mutation shared in at least 2 sequences — these sites with shared mutations are the first step toward the selection of a mutation through selective pressure. Similar counts on baseline Env-gp120 sequences in chronically infected participants in a prior VRC01 infusion study (3) showed on average 59 sites with mutations found once and 76 additional sites with shared mutations, reflecting that the likelihood that VRC01 will select mutations is considerably higher in sequences sampled in chronic infection than in acute-like sequences.

Second, our results support the notion that VRC01 temporarily suppressed viral replication but that VRC01 levels were insufficient for sustained control. Rebound of viremia occurred while serum VRC01 levels showed an average trough of 221 μg/mL, i.e., 50 times higher than IC_80_ values (range 19–96), suggesting that VRC01 concentrations shall be several hundred–fold higher than IC_80_ values for sustained in vivo effect (7 days after infusion: VRC01 levels were 180 times higher than the IC_80_ values [range 34–1006]; the average ratio was 186 in the study by Lynch et al., ref. 3). Although studies in macaques showed viremia suppression until antibody concentrations were 5 μg/mL (16, 17) and VRC01 showed an IC_50_ of less than  50 μg/mL (18), our study emphasizes that IC_50_/IC_80_ values do not translate to therapeutic concentrations in humans, as previously indicated (8). Several factors can help explain why VRC01 serum levels were inadequate. The kinetics of VRC01 penetration into tissues was probably too slow to enable timely neutralization because ART was discontinued the same day VRC01 was initiated. Antibody levels were most likely insufficient in physiologically relevant reservoirs; e.g., in 2 macaques, tissue levels were 10 to 75 times lower than in plasma 24 hours after 3BNC117 administration (19). At rebound, the median serum ID_50_ neutralization titer in our participants was 196 (average = 242 [range = 86–631] (Supplemental Table 3). These values were comparable to the ID_50_ of 91 (95% CI = 55–1153) estimated to achieve 50% protection in a meta-analysis collating SHIV challenge data in the presence of antibodies on 274 animals (20). However, given that our participants were already infected at the time of the ATI, blocking 50% of rebounding infected cells would not be sufficient. The above study showed that the ID_50_ to achieve 95% protection was 685 (95% CI = 319–1471) and 1,958 for 99% protection — an ID_50_ range well above levels in our participants but that may hypothetically be closer to relevant levels for sustained therapeutic effectiveness against a resurgent infection.

Third, using only sequences, we developed a method to calculate the VRC01 epitope distance to VRC01-sensitive strains that tracked with experimentally measured VRC01 sensitivity. This epitope distance metric, when evaluated against more strains, could prove useful as a rapid alternative to in vitro assays.

Finally, since our study is the first to our knowledge to test the impact of VRC01 infusion on acute-like viral populations, our results are pertinent in the context of the antibody-mediated prevention (AMP) trials that are currently testing VRC01 infusion as a strategy to prevent HIV-1 infection. Although blocking the establishment of an infection differs from suppressing a resurgent infection, our findings suggest that, with respect to the AMP trials, a sieve acquisition effect could be observed (with VRC01-resistant sequences more frequent in the treated arm, especially at the highest VRC01 dose) but that postinfection sieving (i.e., the appearance of VRC01-mediated mutations in the founder virus) is less likely.

## Methods

### HIV-1 phylogenetic analysis.

HIV-1 sequences were amplified from plasma RNA as previously described (21).

### In vitro neutralization assays.

RV397 Env-pseudotyped viruses were generated and incubated with RV397 serum or VRC01. Neutralizing serum antibody titers are expressed as the antibody concentration required to achieve 50%/80% neutralization.

### VRC01 epitope distance prediction.

The epitope distance between a virus sequence *X* and a reference sequence *R* was defined as:

*D*(*R*, *X*) = *M*(*R*, *R*) – *M* (*R*, *X*)

*M*(*R*, *X*) = [∑*_i_w_i_* × *Sim*(*R_i_*, *X*i)]/∑*_i_w_i_*

*M*(*R*, *X*) is the distance between *R* and *X*. The distance at the amino acid site *i* between *R* and *X*, *Sim*(*R_i_*, *X_i_*), was calculated using BLOSUM62 (22) matrices; *w_i_* is the weight assigned to epitope site *i* based on the inspection of resolved VRC01-Env complex structures.

### Statistics.

Comparisons between 2 time points were done using Wilcoxon’s signed-rank test and correlations were assessed with Spearman’s coefficients. A *P* value less than 0.05 was considered significant. Analyses were performed in Python and R.

Additional methods are available in Supplemental Methods. Sequences were deposited in GenBank (MT121311–MT121958).

### Study approval.

The randomized, double-blind, placebo-controlled RV397 clinical trial was conducted at the Thai Red Cross AIDS Research Centre in Bangkok, Thailand (1) and approved by institutional review boards at Chulalongkorn University, the Walter Reed Army Institute of Research, and all collaborating institutions. All participants gave written informed consent.

## Author contributions

BD, HB, EMC, NADR, JRM, TAC, JA, ST, and MR conceptualized the study. BD, HB, EMC, NADR, ST, and MR developed methodology. BD, HB, YL, MAM, EMC, RML, LT, A Pagliuzza, NADR, JRM, ESB, MB, AMO, BM, EE, DS, ST, RTB, A Pegu, NC, SP, NP, and MR investigated. MR wrote the original draft of the manuscript. All authors reviewed and edited the manuscript. BD, YL, and HB were responsible for visualization. DJC, CS, EK, TAC, JI, KB, NLM, MLR, and JA designed and conducted the clinical trial. RG, SV, NLM, MLR, JRM, and JA acquired funding.

## Supplementary Material

Supplemental data

## Version 1 03/17/2020

In-Press Preview

## Version 2 05/18/2020

Electronic publication

## Version 3 06/01/2020

Print issue publication

## References

[1] Crowell TA (2019). Safety and efficacy of VRC01 broadly neutralising antibodies in adults with acutely treated HIV (RV397): a phase 2, randomised, double-blind, placebo-controlled trial. Lancet HIV.

[2] Ledgerwood JE (2015). Safety, pharmacokinetics and neutralization of the broadly neutralizing HIV-1 human monoclonal antibody VRC01 in healthy adults. Clin Exp Immunol.

[3] Lynch RM (2015). Virologic effects of broadly neutralizing antibody VRC01 administration during chronic HIV-1 infection. Sci Transl Med.

[4] Bar KJ (2016). Effect of HIV antibody VRC01 on viral rebound after treatment interruption. N Engl J Med.

[5] Shankarappa R (1999). Consistent viral evolutionary changes associated with the progression of human immunodeficiency virus type 1 infection. J Virol.

[6] Keele BF (2008). Identification and characterization of transmitted and early founder virus envelopes in primary HIV-1 infection. Proc Natl Acad Sci U S A.

[7] Ananworanich J (2016). HIV DNA set point is rapidly established in acute HIV infection and dramatically reduced by early ART. EBioMedicine.

[8] Webb NE, Montefiori DC, Lee B (2015). Dose-response curve slope helps predict therapeutic potency and breadth of HIV broadly neutralizing antibodies. Nat Commun.

[9] Doria-Rose NA (2017). Mapping polyclonal HIV-1 antibody responses via next-generation neutralization fingerprinting. PLoS Pathog.

[10] Zhou T (2010). Structural basis for broad and potent neutralization of HIV-1 by antibody VRC01. Science.

[11] Zhou T (2013). Multidonor analysis reveals structural elements, genetic determinants, and maturation pathway for HIV-1 neutralization by VRC01-class antibodies. Immunity.

[12] Stewart-Jones GB (2016). Trimeric HIV-1-Env structures define glycan shields from clades A, B, and G. Cell.

[13] Colby DJ (2018). Rapid HIV RNA rebound after antiretroviral treatment interruption in persons durably suppressed in Fiebig I acute HIV infection. Nat Med.

[14] Sterjovski J (2011). CD4-binding site alterations in CCR5-using HIV-1 envelopes influencing gp120-CD4 interactions and fusogenicity. Virology.

[15] Bai H, Li Y, Michael NL, Robb ML, Rolland M (2019). The breadth of HIV-1 neutralizing antibodies depends on the conservation of key sites in their epitopes. PLoS Comput Biol.

[16] Shingai M (2013). Antibody-mediated immunotherapy of macaques chronically infected with SHIV suppresses viraemia. Nature.

[17] Barouch DH (2013). Therapeutic efficacy of potent neutralizing HIV-1-specific monoclonal antibodies in SHIV-infected rhesus monkeys. Nature.

[18] Wu X (2010). Rational design of envelope identifies broadly neutralizing human monoclonal antibodies to HIV-1. Science.

[19] Shingai M (2014). Passive transfer of modest titers of potent and broadly neutralizing anti-HIV monoclonal antibodies block SHIV infection in macaques. J Exp Med.

[20] Pegu A (2019). A meta-analysis of passive immunization studies shows that serum-neutralizing antibody titer associates with protection against SHIV challenge. Cell Host Microbe.

[21] Rolland M (2012). Increased HIV-1 vaccine efficacy against viruses with genetic signatures in Env V2. Nature.

[22] Henikoff S, Henikoff JG (1992). Amino acid substitution matrices from protein blocks. Proc Natl Acad Sci U S A.

[23] Yoon H (2015). CATNAP: a tool to compile, analyze and tally neutralizing antibody panels. Nucleic Acids Res.

