# Supplementary material for: Neutralizing antibody VRC01 failed to select for HIV-1 mutations upon viral rebound
Source: J Clin Invest. 2020 May 18;130(6):3299–304. doi: 10.1172/JCI134395 (PMC7259993; doi:10.1172/JCI134395)
Supplement: Supplemental data [file jci-130-134395-s394.pdf]

SUPPLEMENTAL INFORMATION

Table of content

F1 Viral load kinetics after treatment interruption and VRC01 infusion p.2

F2 Viral rebound despite VRC01 serum concentrations above 50 µg/mL p.3

T1 Characteristics of RV397 participants p.4

F3 HIV-1 phylogenetic analysis p.5

F4 Divergence from the founder sequence p.5

S4a. Rare HIV-1 mutations identified upon viral rebound following treatment interruption and VRC01 infusion p.6

S4b. HIV-1 mutations identified in individuals with multiple founder variants upon viral rebound following treatment interruption and VRC01 infusion p.7

S4c. Most sequences diverged by less than 2 nucleotide mutations from the HIV-1 founder p.8

F5 Comparison of viral and VRC01 neutralization sensitivity features between HIV-1 acute infection and viral rebound in placebo recipients. p.9

F6 Relationship between Hill coefficients and the time to rebound p.10

F7 Relationship between the size of the reservoir and the time to rebound p.11

F8 Comparison of sequences from control participants who did not receive VRC01 infusion to sequences known to be the most sensitive to VRC01 or CD4 p.12

F9 Differential neutralization with VRC01 or CD4-Ig for sequences with D or N at position 279 p.13

T2 Amino acid frequencies across different HIV-1 subtypes at position 279 p.14

F10 Env complex structures with CD4 and VRC01 p.15

F11 Lack of relationship between the predicted VRC01 epitope distance and time to rebound in 13 participants who did not receive VRC01 infusions p.16

F12 Relationship between the predicted VRC01 epitope distance and experimentally defined VRC01 sensitivity in 17 RV397 participants p.17

T3 Summary table with IC50/80 and ID50/80 neutralization titers p.18

Supplemental methods p.19

Supplemental acknowledgments – RV397 Study Group p.22

Supplemental references p.23

Suppl. Figure 1 Viral load kinetics after treatment interruption and VRC01 infusion

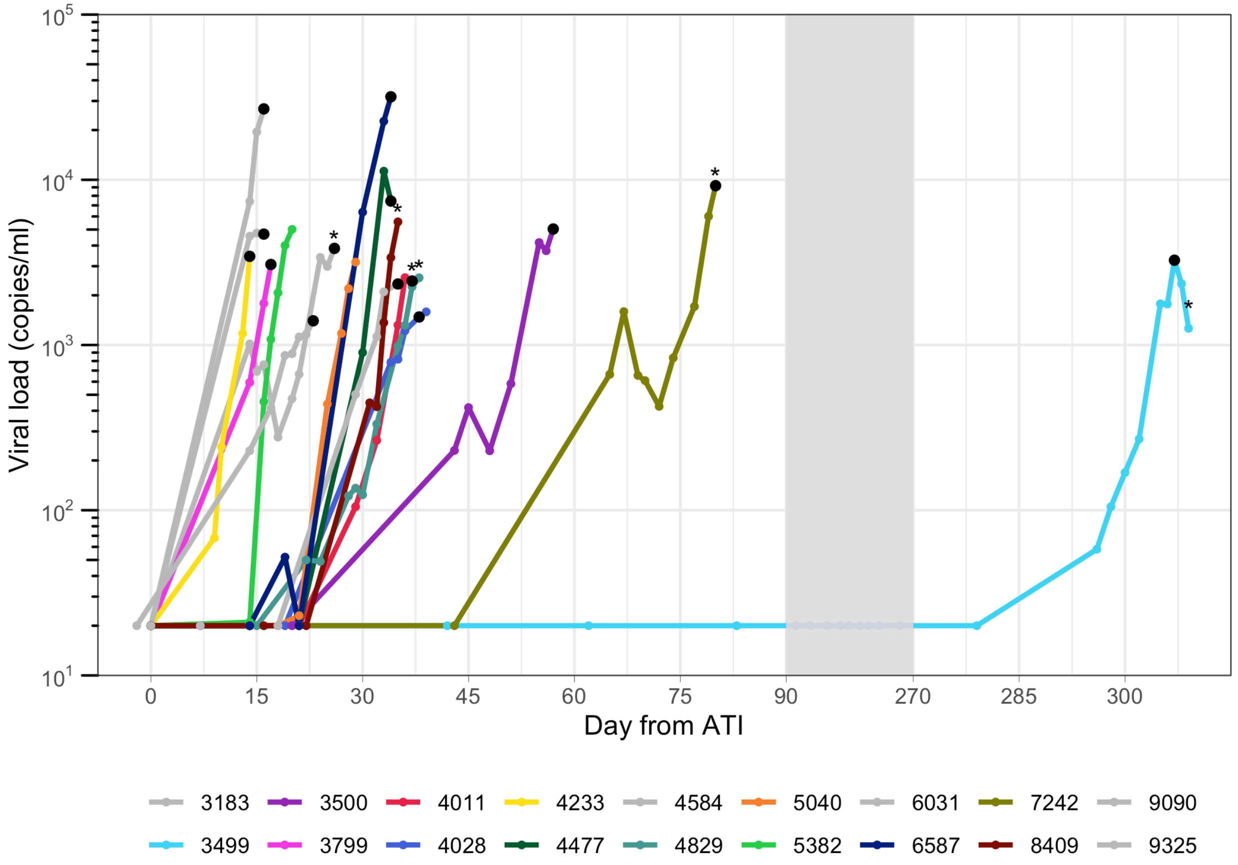

Viral load kinetics after treatment interruption and VRC01 infusion. Viral load measurements are figured in a different color for each treated participant and in grey for placebo recipients. The time point used for sequencing at viral rebound is marked with a black dot. Asterisks mark infections by non CRF01_AE viruses. The x axis is rescaled between days 90 and 270.

Suppl. Figure 2 Viral rebound despite VRC01 serum concentrations above 50 µg/mL

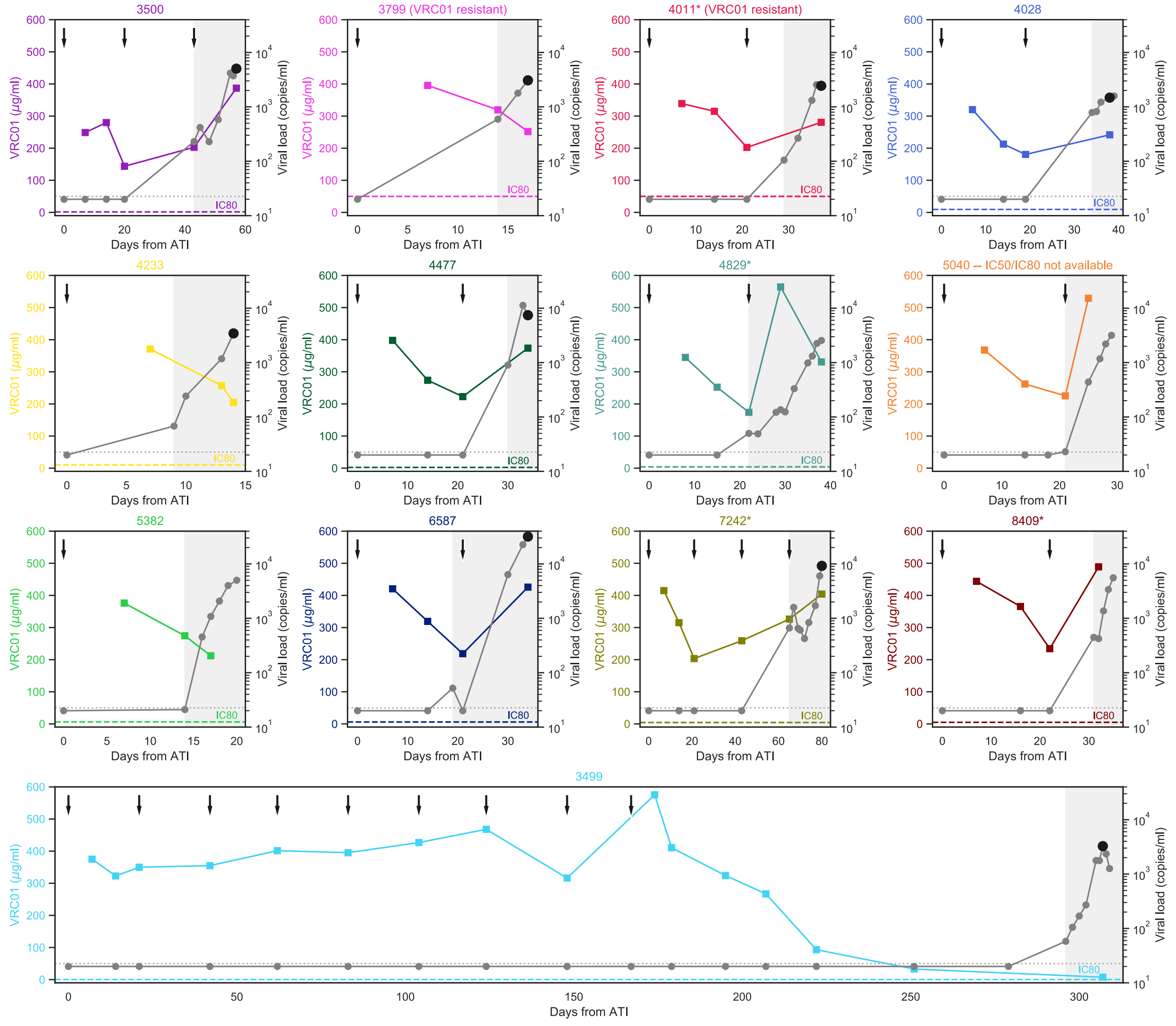

Viral rebound despite VRC01 serum concentrations above 50 µg/mL. Viral load measurements are figured in grey with a shaded grey area to indicate the viral rebound period (> 20 copies/mL); the time point used for sequencing is marked with a black dot. VRC01 infusions (40 mg/kg, every three weeks) are indicated with black arrows. VRC01 serum levels are figured in a different color for each treated participant. Estimated IC80 values are represented with a colored dotted line; a gray dotted line figures the target trough of 50µg/mL. Env-pseudotyped viral strains from all participants except 3799 and 4011 were sensitive to VRC01 in in vitro assays (**Figures 2, 3**).

Suppl. Table 1 Characteristics of RV397 participants

Details on the participants and the sequencing data obtained at HIV-1 diagnosis and upon rebound are provided in the table below.

					Diagnosis		Viral rebound				
ID	VRC01 infusion	Fiebig stage	Years on ART	Days to rebound	VL	n genomes	VL	n pol	n env	Subtype	Founder
3183	0	2	3.8	14	426,027	10	3,845	15	15	B	Single
3499	1	3	3.1	296	3,811,036	10	1,769	15	15	AE/B	Multiple
3500	1	1	2.5	43	58,434	10	5,038	15	15	01_AE	Single
3799	1	2	3.6	14	3,260,216	10	3,072	15	15	01_AE	Single
4011	1	3	6.6	29	913,670	10	2,440	15	15	B	Single
4028	1	3	2.3	34	476,362	4	1,478	15	15	01_AE	Single
4233	1	2	3.6	9	>10M	10	3,440	15	15	01_AE	Single
4477	1	2	4	30	3,669,800	10	7,433	15	15	01_AE	Single
4584	0	3	2.6	14	301,811	10	4,691	16	15	01_AE	Multiple
4829	1	3	2.9	22	26,316	10	2,552	0	0	B	Multiple
5040	1	2	2.4	21	229,991	10	439	0	0	01_AE	Single
5382	1	3	4.5	14	674,135	10	1,083	0	0	01_AE	Single
6031	0	2	2.7	29	1,542,886	10	2,341	15	15	01_AE	Single
6587	1	2	2.4	19	437,266	10	31,807	15	15	01_AE	Single
7242	1	2	5.2	65	708,317	20	9,212	15	18	AE/B/C	Multiple
8409	1	2	2.3	31	173,497	10	423	0	0	B	Single
9090	0	2	2.3	14	>10M	10	26,865	15	15	01_AE	Single
9325	0	3	3.1	14	1,038,623	10	1,401	15	15	01_AE	Single

Suppl. Figure 3 HIV-1 phylogenetic analysis

Participants were diagnosed at Fiebig stages I to III and the median viral load at the time of sequencing before ART was 691,226 copies/mL (range: 26,313 - >10,000,000). After treatment interruption, plasma samples were collected a median of 7 days (range: 2-15) after the first viral rebound measurement over 20 copies/mL; at that time, the median viral load for the 14 participants successfully sequenced was 3,643 copies/mL (range: 1,401 - 31,807); for the four participants that failed sequencing, the median viral load was 723 copies/mL (range 423-2,552). Most participants were infected with single HIV-1 founders represented by monophyletic viral populations (14 of 18). As expected in Thailand, most infections corresponded to CRF01_AE viruses (12 of 18) and CRF01_AE containing recombinants (n = 2), while four infections corresponded to subtype B. The complex viral population in participant 7242 comprised multiple founders with recombinants of CRF01_AE and subtypes B and C, with subtype B limited to the 3' half of the genome. Hence, phylogenetic trees based on *env* showed that sequences from 7242 were polyphyletic while *pol* sequences all belonged to a monophyletic taxon.

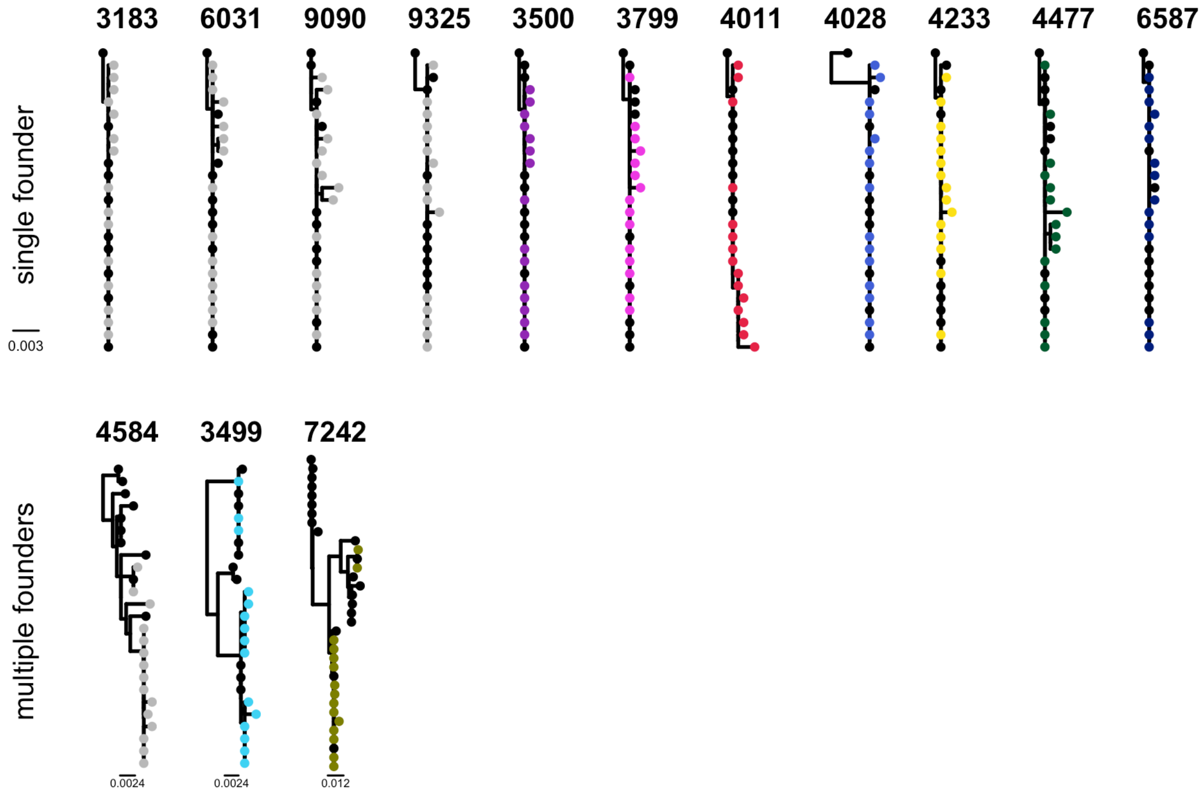

HIV-1 *pol* sequences sampled at HIV-1 diagnosis and upon viral rebound were intermingled in phylogenetic trees. Maximum likelihood trees were reconstructed based on *pol* sequences obtained from plasma samples collected in acute HIV-1 infection (when ART was initiated) and upon viral rebound (following simultaneous VRC01 infusion and treatment interruption, after an average of three years on ART). Sequences derived in acute infection are displayed in black, those sampled upon viral rebound are in colors. Trees with light gray dots indicate placebo recipients. The horizontal bar represents the number of substitutions per site; the scale differs for infections with single or multiple founders. Four participants had *env* sequences corresponding to subtype B (3183, 4011) or a CRF01_AE containing-recombinant (3499, 7242).

Suppl. Figure 4a   Divergence from the founder sequence

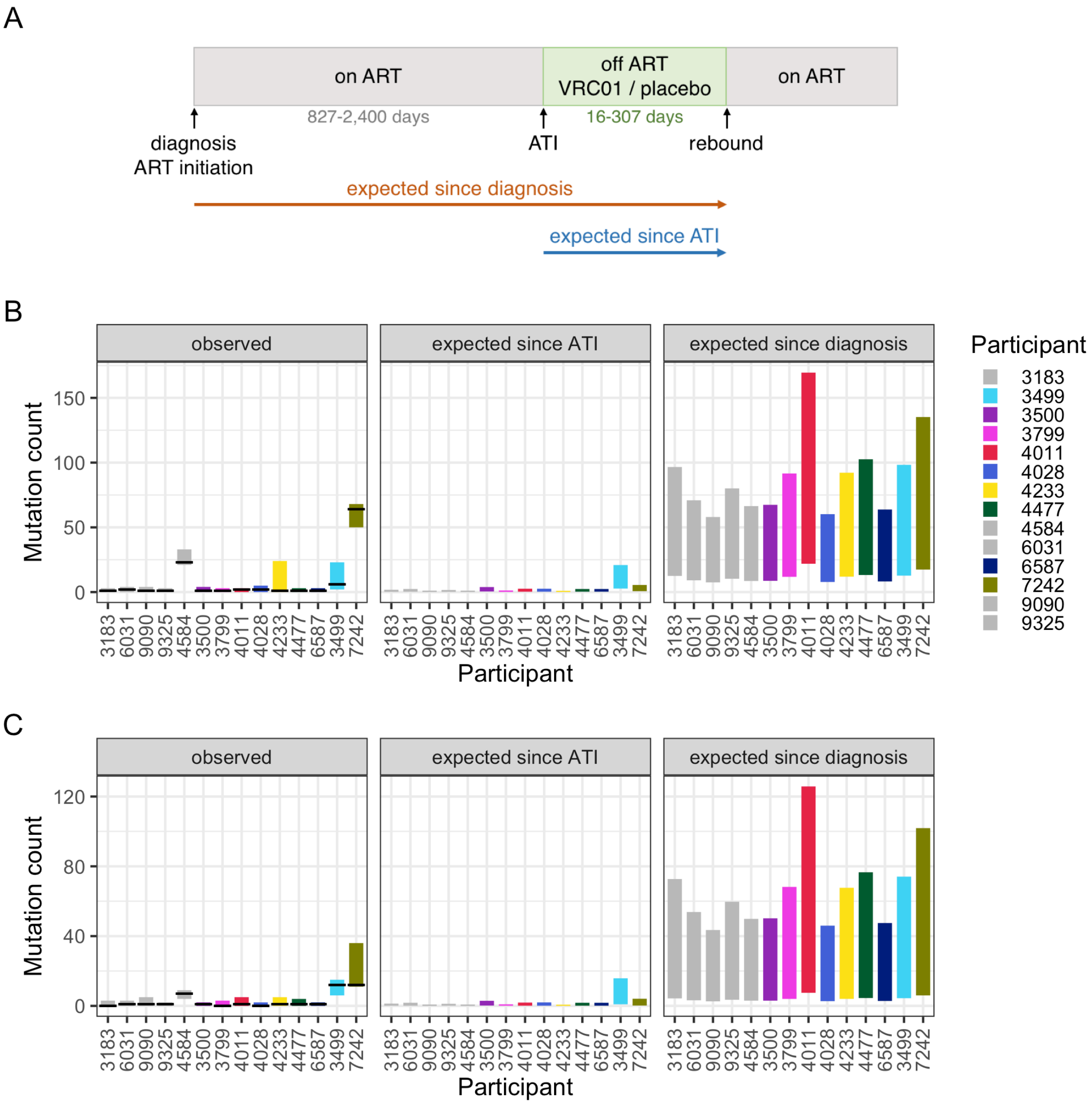

S4a. Rare HIV-1 mutations identified upon viral rebound following treatment interruption and VRC01 infusion. Panel A shows the time course of the study. Mutations were identified by comparing the nucleotide sequences sampled upon viral rebound to the founder consensus derived from sequences sampled in acute HIV-1 infection. The number of mutations in *env* and *pol* is reported in panels B and C, respectively. The median (line) and range of mutations observed in RV397 sequences (left panel) is compared to the number of mutations that would be expected to have occurred since treatment cessation (middle panel) or since the beginning of the infection (right panel) in a given participant. Expected numbers were calculated based on an independent dataset comprised of acutely infected, untreated individuals (Rolland et al., PLoS Pathogens, in press). Mutations in each sequence were counted based on the consensus derived from all sequences obtained at HIV-1 diagnosis. Participants 3499, 7242 and 4584 were infected with multiple HIV-1 founder variants; mutation counts against the different variants are shown in S4b. There was no significant relationship between the number of mutations and the time to sequencing or to ART re-initiation after the ATI: Rho≤0.26, p≥0.38).

S4b. HIV-1 mutations identified in individuals with multiple founder variants upon viral rebound following treatment interruption and VRC01 infusion. Mutations were identified in *pol* and *env* nucleotide sequences sampled upon viral rebound by comparison with the founder consensus sequences derived from extant sequences sampled in acute HIV-1 infection. Participants 3499, 7242 and 4584 were infected with multiple HIV-1 founder variants and different founder consensus sequences were inferred to represent the different founders in each participant's viral population. The average number and range (in parentheses) of mutations observed in RV397 sequences is compared to the number of mutations that would be expected to have occurred since either the beginning of the infection or since treatment cessation in a given participant. Expected numbers were calculated based on an independent dataset comprised of acutely infected, untreated individuals (Rolland et al., PLos Pathogens, in press).

	Participant	Observed (median, range)	Expected since ATI	Expected since diagnosis
Env	4584	26 (1-31)	0.1 - 1.1	8.6 - 66.4
	3499	4 (0-10)	2.7 - 20.9	12.7 - 98.3
	7242	43 (29-47)	0.7 - 5.5	17.4 - 135.2
Pol	4584	7 (5-10)	0 - 0.8	3 - 49.9
	3499	1 (0-4)	0.9 - 15.8	4.4 - 74.1
	7242	26 (15-32)	0.2 - 4.1	6 - 101.9

S4c. Most sequences diverged by less than 2 nucleotide mutations from the HIV-1 founder.

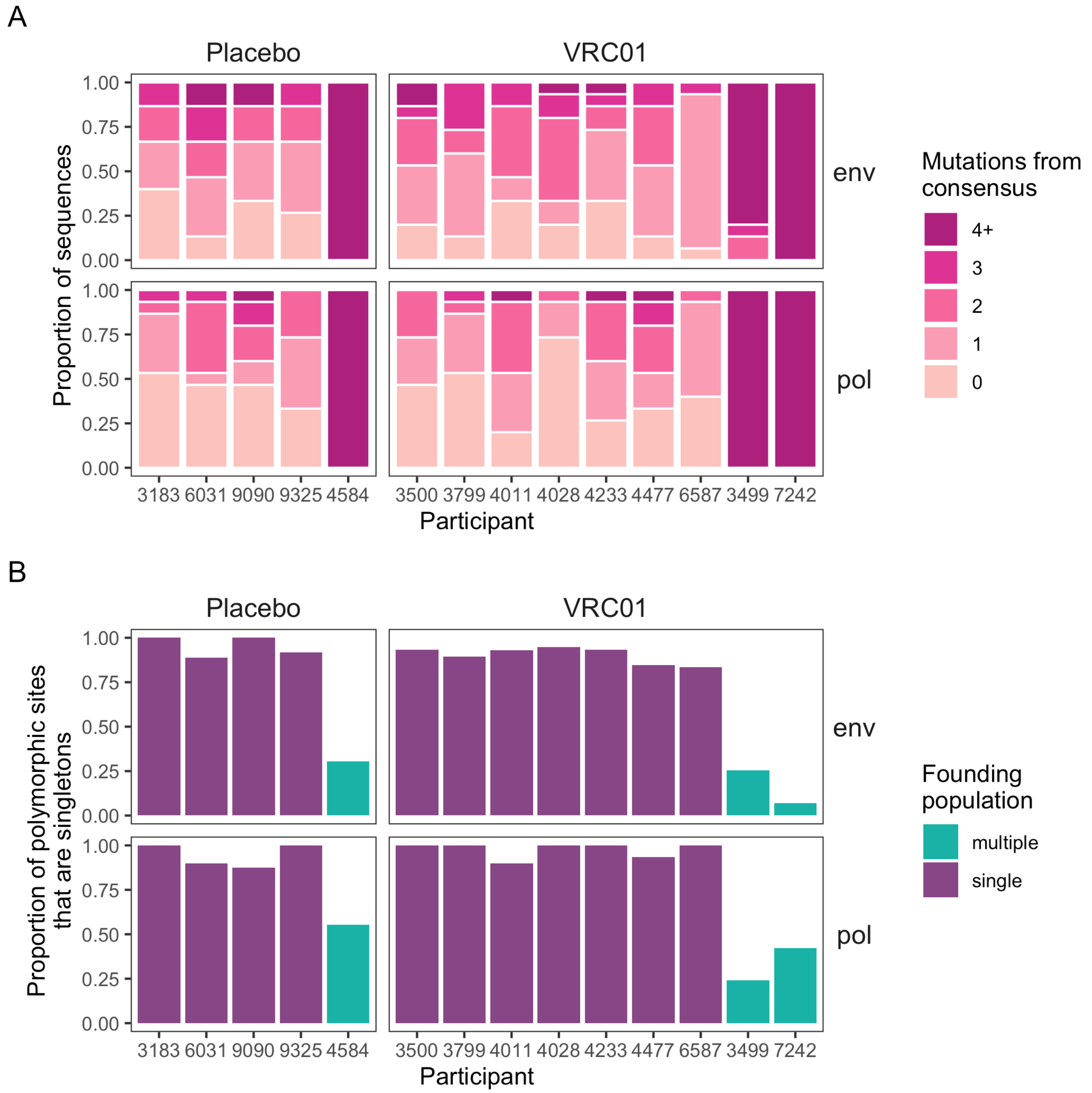

Most sequences diverged by less than 2 nucleotide mutations from the HIV-1 founder. Each participant's rebound sequences were compared to their corresponding founder variant to count the number of mutations from the founder. (A) The proportion of rebound sequences with a given number of mutations from the founder consensus are reported for each participant. (B) The proportion of polymorphic sites that represent mutations found in a single sequence or that are shared across sequences from a participant. Most polymorphic sites among sequences from a participant corresponded to singletons, and only a small proportion of shared mutations were identified (here, shared mutations could reflect selection). For infections with multiple founders, sequences within a founder lineage share mutations distinguishing that founder from other founder(s), and therefore have fewer singleton sites by definition.

Suppl. Figure 5 Comparison of viral and VRC01 neutralization sensitivity features between HIV-1 acute infection and viral rebound in placebo recipients

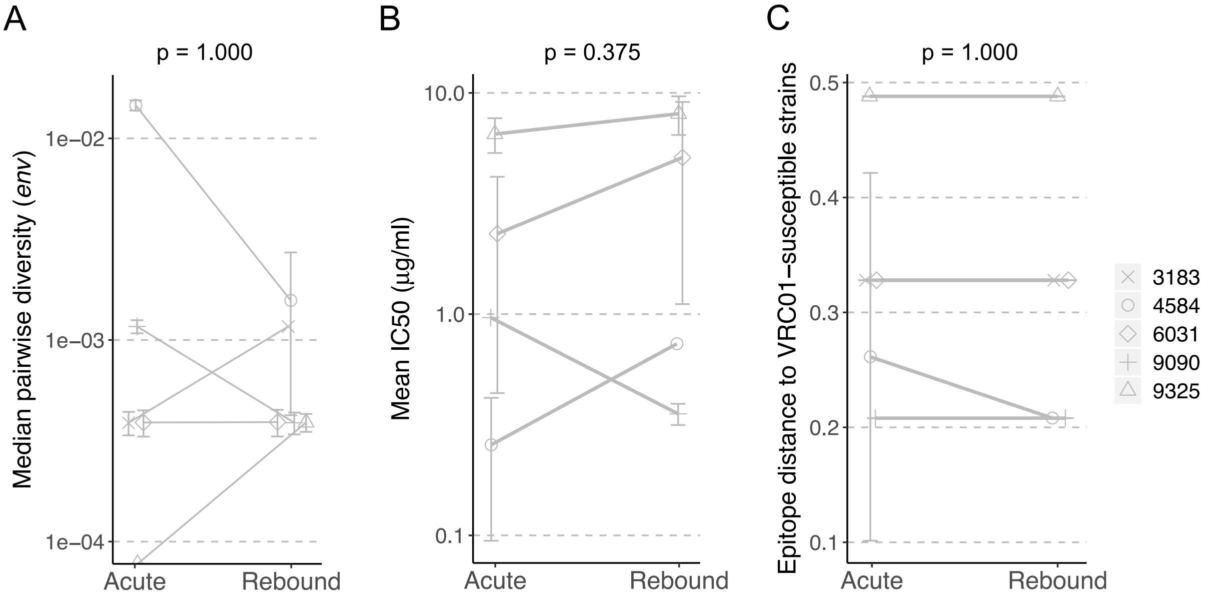

Sequence diversity, epitope distance and VRC01-neutralization sensitivity values were similar between HIV-1 acute infection and viral rebound in placebo recipients. Comparisons showed no significant difference across time points for the median pairwise diversity across *env* nucleotide sequences from each placebo participant (panel A), VRC01-specific IC50 values (panel B) and VRC01 epitope distances (panel C). Wilcoxon signed-rank tests were used for comparisons.

Suppl. Figure 6   Relationship between Hill coefficients and the time to rebound.

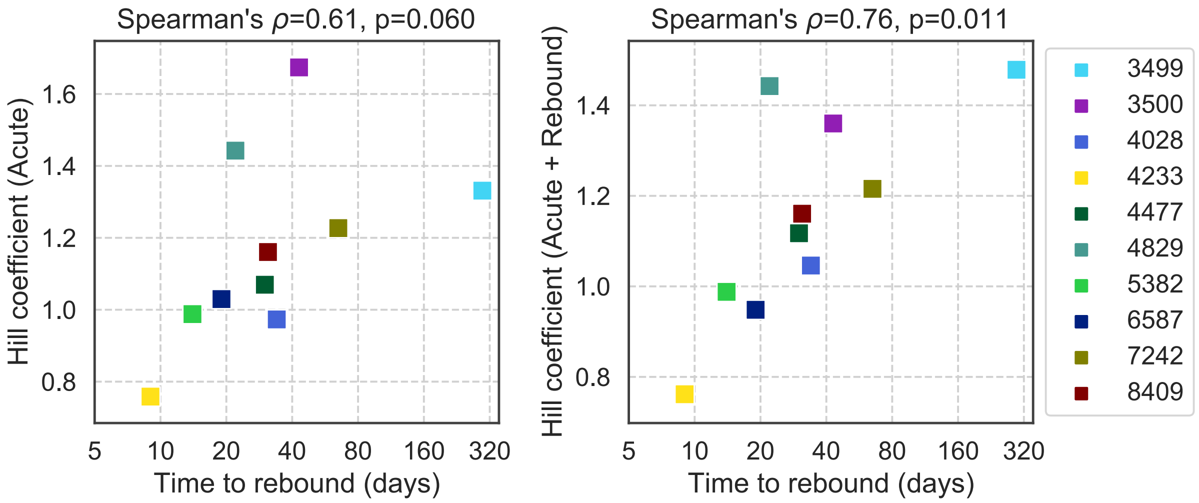

Higher Hill coefficients were associated with delayed viral rebound. Hill coefficients correspond to the slopes of the dose-response (VRC01-neutralization) curves for each participant. They are considered to predict better than IC50/IC80 values the inhibition potency of an antibody at clinically relevant levels. The left panel corresponds to Hill coefficients based on data from sequences from acute infection only; the panel on the right includes all the data from the acute and rebound time points.

Suppl. Figure 7 Relationship between the size of the reservoir and the time to rebound.

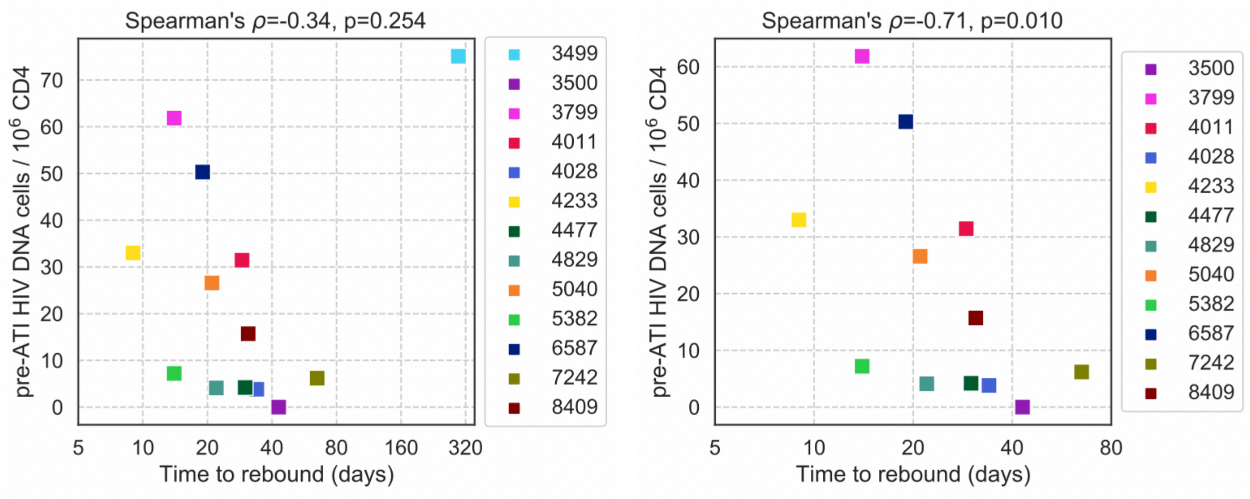

No relationship between the reservoir size and time to rebound except if participant 3499 was excluded. The left panel shows no significant association. The panel on the right excludes the participant who suppressed viremia for 296 days (3499) and shows that a larger reservoir size before ATI was associated with a faster time to rebound. The size of the reservoir was measured as the number of cells with HIV DNA per million CD4 cells.

Suppl. Figure 8 Comparison of sequences from control participants who did not receive VRC01 infusion to sequences known to be the most sensitive to VRC01 or CD4

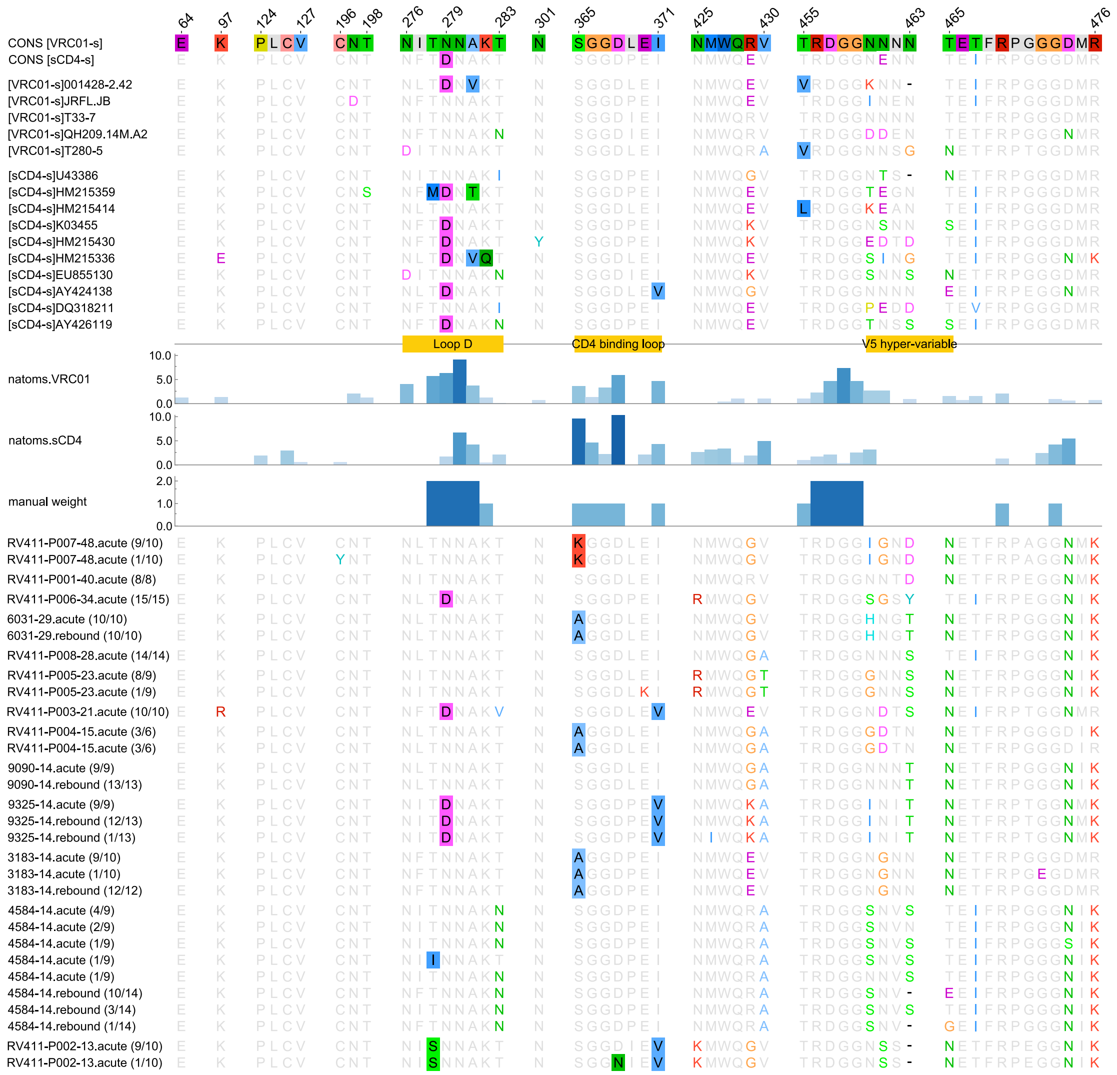

Comparison of sequences from control participants who did not receive VRC01 infusion to sequences known to be the most sensitive to VRC01 or CD4. The top sequences correspond to the consensus residues found in sequences that were experimentally identified as the most sensitive to VRC01 (1) and soluble CD4 (2). The importance of specific residues in the interaction with Env (measured by the number of VRC01 or sCD4 atoms that contact a given residue) is figured below with darker colors corresponding to more influential residues. The core and rim epitope sites had a weight of 2 and 1, respectively (details in the supplementary method). Sequences from RV397 participants from the placebo group (n = 5) and participants in an ATI study with no intervention (RV411, Colby et al., 2018) (n = 8) are labeled with a suffix corresponding to the number of days that had elapsed when they rebounded. Numbers in parentheses correspond to the number of sequences with the given epitope motif and the total number of sequences sampled from a participant. Participants are ordered from longest to shortest time to rebound (from 296 to 9 days).

Suppl. Figure 9 Differential neutralization with VRC01 or CD4-Ig for sequences with D or N at position 279

Inspection of Env structures illustrates that the presence of D at position 279 could be favored by CD4 (**S12**). One hypothesis is that it could facilitate HIV-1 entry/infection, meaning that VRC01 competition might be less effective thereby reducing the VRC01-mediated viral containment. We compared the CD4-Ig sensitivity between individuals with either a D or an N at position 279: while we noted that viruses with D at position 279 tended to be more CD4-sensitive than those with N at 279, this difference was not significant; in contrast, viruses with D at 279 were significantly more resistant to VRC01 (p = 0.016).

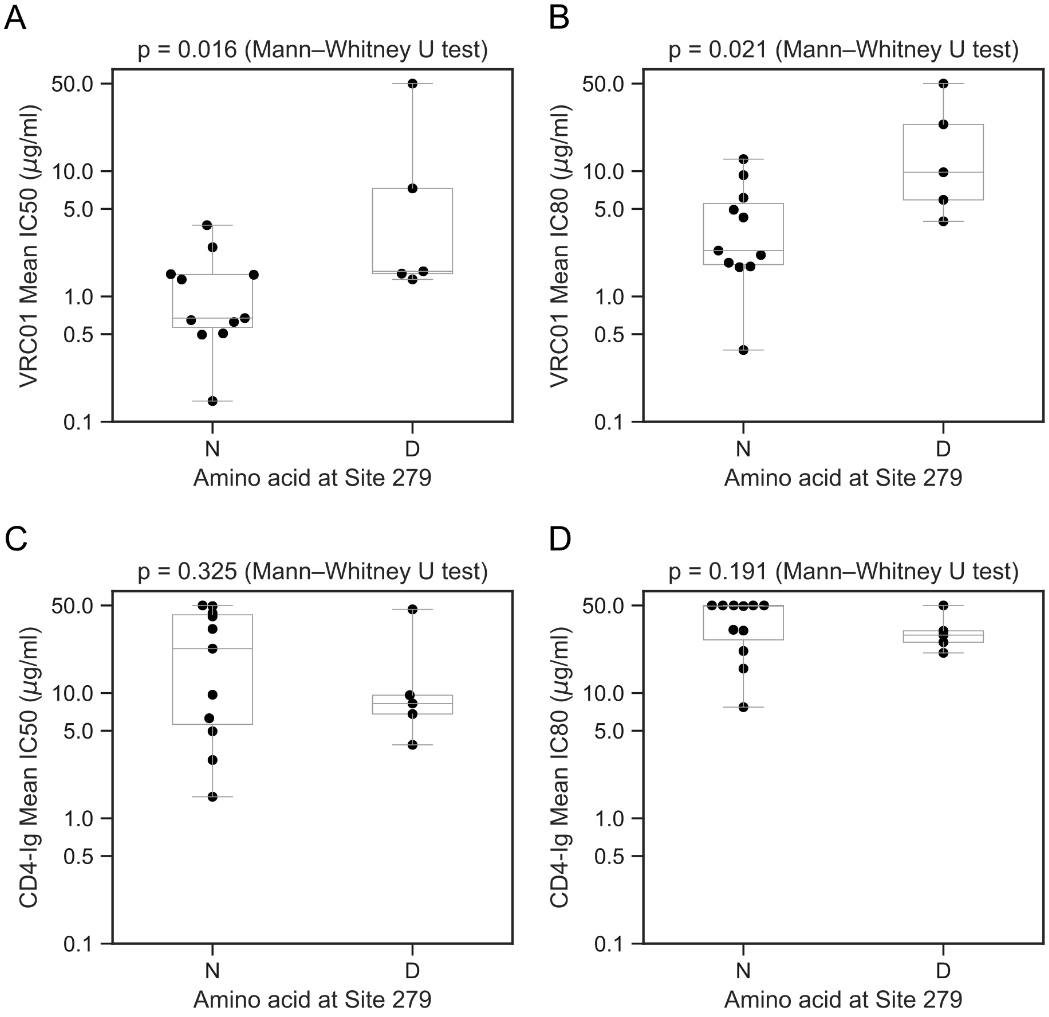

Differential neutralization with VRC01 or CD4-Ig for participant-derived pseudoviruses. IC50 and IC80 values were measured for VRC01 and CD4-Ig against participant-derived pseudoviruses. The participants' pseudoviruses were compared based on the presence of N or D at position 279 in Env.

Suppl. Table 2 Amino acid frequencies across different HIV-1 subtypes at position 279
Alignments of Env sequences were prepared by curating publicly available sequences to retain only those that had correct open reading frames, no hypermutations, no ambiguous residues and information on their sampling dates and locations. Amino acid frequencies are shown in percentages, based on sequence alignments that include between 116 and 1,184 sequences (subtype A1: n = 203, subtype B: n = 1,035, subtype C: n = 1,184, subtype D: n = 116, CRF01_AE: n = 577). N and D are the two most frequent AA found at position 279 for each dataset; the third most frequent AA differs across subtypes, its frequency is shown in parentheses.

Position 279	A1	B	C	D	CRF01_AE
**N**	62.1	47.8	48.8	71.6	64.1
**D**	33.0	49.8	47.6	24.1	34.3
**Third AA**	S (2.0)	E (1.2)	S (1.2)	S (3.5)	E (0.7%)

Suppl. Figure 10 Env complex structures with CD4 and VRC01

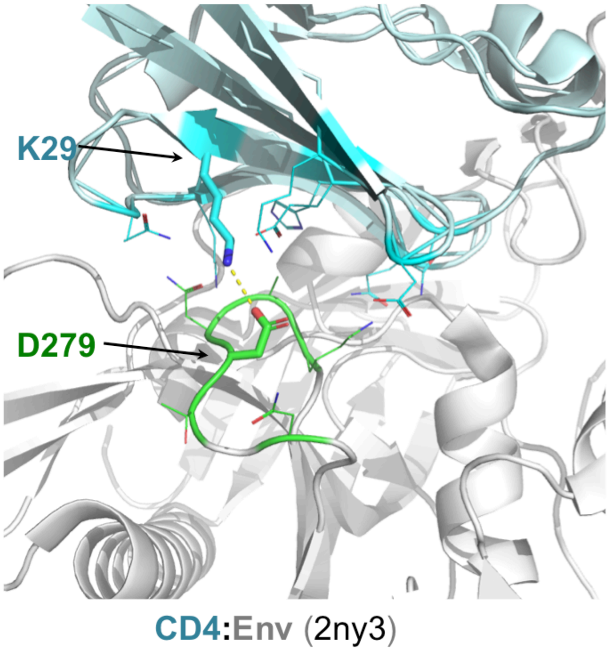

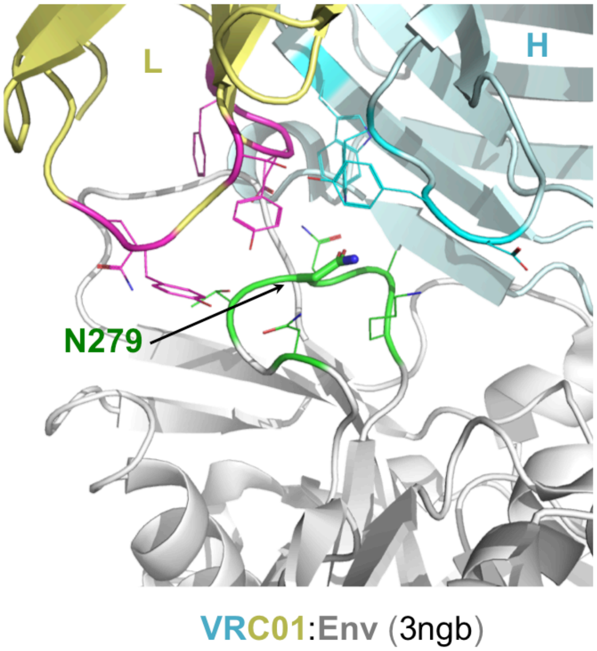

Env complex structures with CD4 and VRC01. The structures 2ny3 and 3ngb are represented to reflect potential differences associated with the presence of the residues N or D at position 279 for CD4 and VRC01 binding. A putative salt bridge between Env position D279 and residue K29 of CD4 is indicated by a dotted yellow line.

Suppl. Figure 11 Lack of relationship between the predicted VRC01 epitope distance and time to rebound in 13 participants who did not receive VRC01 infusions

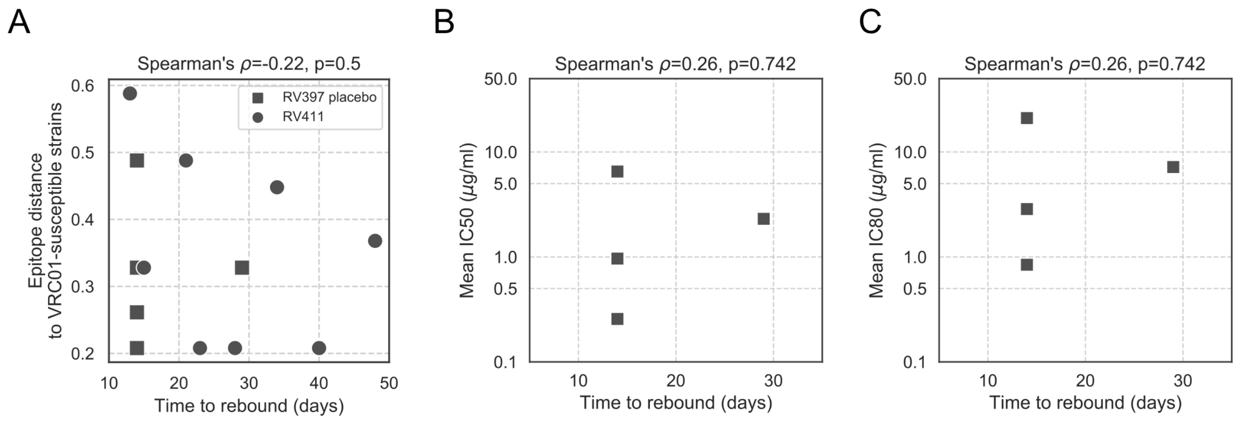

Lack of relationship between the predicted VRC01 epitope distance and time to rebound in 13 participants who did not receive VRC01 infusions. There was no association between VRC01 epitope distances (predicted *in silico* from each participant's sequences) and the time to viral rebound when the comparison was done using participants from the placebo group (n = 5) and participants in an ATI study with no intervention (RV411, Colby et al., 2018) (n = 8) (panel A). Panels B and C show the IC50 and IC80 values for placebo recipients in R397, respectively.

Suppl. Figure 12 Relationship between the predicted VRC01 epitope distance and experimentally defined VRC01 sensitivity in 17 RV397 participants

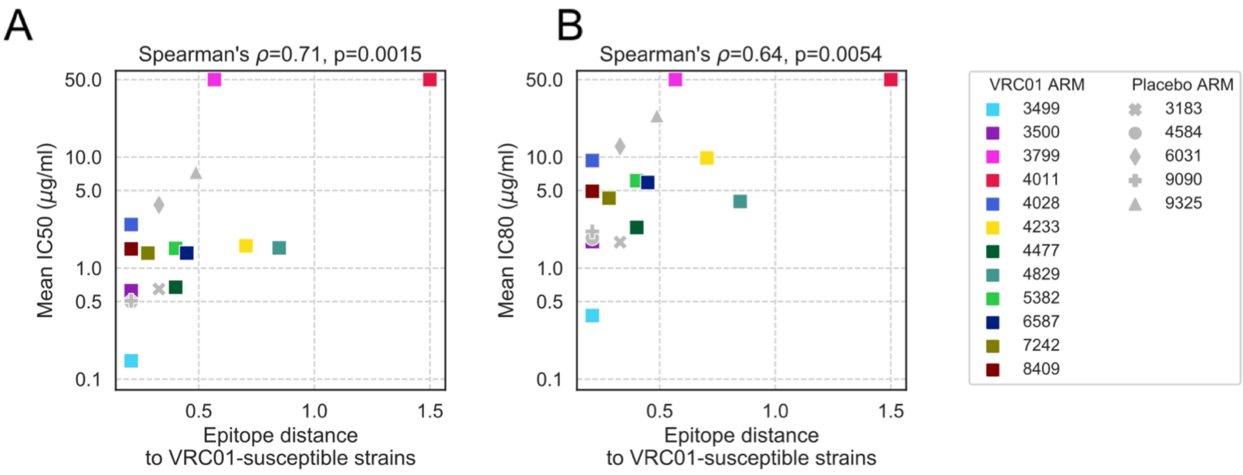

Relationship between the predicted VRC01 epitope distance and experimentally defined VRC01 sensitivity in 17 RV397 participants. Association between VRC01 epitope distances (predicted *in silico* from each participant's sequences) and experimentally defined VRC01-specific IC50 (panel A) and IC80 (panel B) values. Participants who received VRC01 infusions are figured with colored symbols and placebo recipients are in grey.

Suppl. Table 3 Summary Table with IC50/80 and ID50/80 neutralization titers

VRC01 sensitivity in relation to serum antibody concentrations at the time of viral rebound for the 13 participants who received VRC01 infusions.

ID	Time to rebound (days)	Rebound VL (copies/ml)	Mean IC50 (ug/ml)	Mean IC80 (ug/ml)	Hill coefficient (a)	VRC01_level at rebound (ug/ml) (b)	ID50 titer at rebound	ID80 titer at rebound
3499	296	58	0.15	0.37	1.48	12.5	85.6	33.6
3500	43	230	0.63	1.73	1.36	202.8	324.0	116.9
3799	14	593	50	50	NA	319.4	NA	NA
4011	29	105	50	50	NA	358.4	NA	NA
4028	34	787	2.47	9.29	1.05	289.2	117.1	31.1
4233	9	68	1.59	9.8	0.76	332.9	209.4	34.0
4477	30	900	0.67	2.32	1.12	423.7	630.5	182.3
4829	22	50	1.52	3.98	1.44	173.9	114.4	43.7
5040	21	23	NA	NA	NA	225.2	NA	NA
5382	14	21	1.51	6.13	0.99	274.4	181.7	44.8
6587	19	52	1.37	5.9	0.95	247.3	180.5	41.9
7242	65	664	1.37	4.27	1.22	326.2	238.1	76.3
8409	31	447	1.49	4.92	1.16	502.6	337.3	102.1

(a) Hill coefficient of dose-response curve estimated as log(0.8/0.2) / log(IC80/IC50).

(b) Experimental (if measured) or estimated VRC01 level at the time virus rebound (>20 copies/mL) was detected

Supplemental methods

HIV-1 sequencing

HIV-1 pro-RT (HXB2 corresponding numbers 2071-3870) and envelope gp160 (5956-9089) were amplified from plasma RNA following our previously described single genome amplification strategy (3). A nested PCR strategy was employed with primers specific for CRF01_AE and subtype B. cDNA synthesis was performed using JL68Rv2 (5'-CTTCTTCCTGCCATRKGARA 3') for pro-RT and oligodT20 for gp160. First round primers were pol_RT2071F (5'-GARAGRCAGGCTAATTTTTTAGG-3') and pol_G.AGrev (5'TRTCTACTTGTTCA TTTCCYCC-3') for pro-RT; polK3 (5'-TAAARYTAGCAGGAAGATGGCCAGT-3') and uninef7 (5'-GCACTCAAGGCAAGCTTTATTGAGGCTT-3') for gp160. Second round primers were polRT2071F (5'-GARAGRCAGGCTAATTTTTTAGG-3') and polRT3870R (5'-CTATTRGCTGCCC CATCTA 3') for pro-RT, JL68 (5'-CTTAGGCATCTCCTATGGCAGGAAGAAG-3') and JL89 (5'-TCCAGTCCCCCCTTTTCTTTTAAAAA-3') for gp160.

HIV-1 sequence analysis

Participant sequences were aligned with MAFFT (4) before manual editing using Aliview (5). Maximum likelihood phylogenetic trees were derived using FastTree 2.1.10 compiled with double precision (6). Mutation counts were obtained using InSites as implemented in DIVEIN (7) (https://indra.mullins.microbiol.washington.edu/DIVEIN).

HIV-1 circulating *env* sequences were retrieved from the Los Alamos HIV Database (http://www.hiv.lanl.gov/) on 06/03/2018 for subtypes A1, B, C, D and CRF01_AE. The datasets were curated to retain only independent sequences (one sequence per individual) with a correct open reading frame; we removed sequences with no time stamp or subject identifier, with frame shifts, stop codons or ambiguous residues, and with evidence of hypermutation or recombination. Alignments were edited as described above.

The divergence of any sequence away from the founder consensus was calculated by fitting a linear regression of the form: $y = \beta_0 + \beta_1 x$, where x is the sampling time in days since the first positive RNA test and y is the divergence from the consensus. The gradient of the regression line, β_1, estimated the substitution rate in substitutions per site per day; this gradient was derived from an independent training data set that included 30 sequences sampled in the first six months of infection from 12 individuals from the RV217 cohort infected with CRF01_AE viruses (Rolland et al., in press) (8). Using the minimum and maximum substitution rates from the training set, the expected number of substitutions was calculated as $r * d * l$, where r is the estimated rate from the training set, d is the number of days to consider evolution and l is the sequence length. Two possible periods of evolution were considered: 1) from ART interruption to rebound, counted as the number of days from ART interruption to sequencing (not counting days after ART re-initiation); and, 2) from sequencing at HIV-1 diagnosis to sequencing post-rebound, assuming replication during ART.

HIV-1 Env-pseudotyped virus production

Plasma-derived *env* genes were cloned into vector pVRC8400. Pseudoviruses for use in TZM-bl neutralization assays were produced in 293T cells (HEK 293T/17, ATCC® CRL-11268™) by cotransfection of a pSG3ΔEnv backbone plasmid and the full HIV-1 Env gp160-encoding plasmid. Briefly, $2X10^6$ cells in 20ml cDMEM (DMEM supplemented with 10% fetal calf serum and 1X penicillin/streptomycin) were seeded in T75 flasks the day prior to cotransfection. For transfection, 40 μL of FuGene 6 reagent (Promega, Fitchburg, WI) was diluted into 800 μL of room temperature Opti-MEM I reduced serum medium (Thermo Fisher, Waltham, MA), followed by addition of 10 μg of pSG3ΔEnv backbone plasmid. 3.3 μg of HIV Env plasmid was then added to the mixture, mixed, and incubated for 30 min at room temperature. The transfection mixture was then added to media of previously seeded 293T cells in the T75 flask and then distributed evenly on cells. The following day, media was replaced with 20ml fresh cDMEM. Virus was harvested the following day by filtering cell supernatants with 0.45 mm Steriflip units (EMD Millipore) and aliquoted.

In vitro neutralization assays

Ten μL of 5-fold serially diluted mAbs in cDMEM were incubated with 40 μl of diluted HIV-1 Env-pseudotyped virus and incubated for 30 min at 37°C in a 96-well black tissue culture plate. 20 μL of TZM-bl cells (NIH AIDS Reagent Program, Catalog number: 8129)(10,000 cells/well) with or without 70 mg/ml DEAE-Dextran (10 mg/mL final concentration) was then added and incubated overnight at 37°C. Each experiment plate also had a column of cells only (no Ab or virus) and a column of virus only (no Ab) as controls for background TZM-bl luciferase activity and maximal viral entry, respectively. The following day, all wells received 100 μL of fresh cDMEM and were incubated overnight at 37°C. The following day, 50 μl of Steadylite Plus luciferase reagent (PerkinElmer, Inc., Waltham, MA) was added to each well, and plates were shaken at 600RPM for 15 min. Luminometry was performed on a SpextraMax L luminometer (Molecular Devices, San Jose, CA). Percent neutralization was determined by calculating the difference in average RLU between virus-only wells (cells + virus column) and test wells (cells + serum/Ab sample + virus), dividing this result by the average RLU of virus-only wells (cells + virus column) and multiplying by 100. Background is subtracted from all test wells using the average RLU from the uninfected control wells (cells only column) before calculating the percent neutralization. Neutralizing serum antibody titers are expressed as the antibody concentration required to achieve 50% neutralization and calculated using a dose-response curve fit with a 5-parameter nonlinear function.

Estimation of VRC01 decay rates

VRC01 decay rates were estimated as the slope of the linear regression line of VRC01 concentrations over time. We calculated decay rates after the first VRC01 infusion or after the last VRC01 infusion before rebound.

Estimation of Hill coefficients

The dose-response curve corresponding to the VRC01 neutralization of pseudoviruses derived from RV397 participants' sequences was assumed as a Hill-curve. The Hill coefficient of a neutralization curve, which characterizes how neutralization increases with higher VRC01 concentrations, was estimated as log[0.8/(1-0.8)] / log(IC80/IC50), based on experimentally determined IC50 and IC80 values. Higher Hill coefficients indicate increased effectiveness.

VRC01 epitope distance prediction

The epitope distance between a virus sequence X and a reference sequence R was defined as:

$$\mathrm{D(R,X)} = \mathrm{M(R,R)} - \mathrm{M(R,X)}$$

$$\mathrm{M(R,X)} = [\textstyle\sum_{\mathrm{i}} \mathrm{w_i} \cdot \mathrm{Sim(R_i, X_i)}\,]/\textstyle\sum_{\mathrm{i}} \mathrm{w_i}$$

M (R, X) is the distance between R and X. The distance at the amino acid site i between R and X, Sim (R_i, X_i), was calculated using BLOSUM62 (9) matrices; w_i is the weight assigned to epitope site i.

The weight of each epitope site was assigned based on the inspection of resolved VRC01:Env complex structures (PDB code: 3ngb (10), 4lst (11), 5fyj (12) and 5fyk (12)). Any Env site with heavy atoms within 4 Å of VRC01 in the complexes was considered as an epitope site. The weight of each epitope site was manually assigned, such that the core sites in the epitope (T278, D279, N280, A281, R456, D457, G458, G459) had a weight of 2, the rim sites that loosely contacted VRC01 (K97, G197, G198, N276, T430, A460, N461, T463, N465, T467) had a weight of 0 and sites in-between (K282, S365, G366, G367, D368, I371, T455, R469, G473) had a weight of 1; amino acid numbering corresponds to the PDB structure 3ngb.

The most susceptible strains to VRC01 were used as references and were selected as the five sequences with the lowest IC50 values in a panel of 136 viruses (1).

Statistical Analysis

Data analysis, visualization and statistical testing were performed in Python and R (https://www.r-project.org/) environment (13-22). Statistical details of analyses can be found in the main text and figure captions where applicable; significance was established at $p < 0.05$. A link to the data archive and code to reproduce the analysis is provided below.

Data Availability

HIV-1 sequences were deposited in GenBank under accession numbers: MT121311-MT121958.

Supplemental acknowledgments

RV397 Study Group

Julie Ake, Siriwat Akapirat, Diane Bolton, Phillip Chan, Sararut Chanthaburanun, Nitiya Chomchey, Nampueng Churikanont, Peter Dawson, Netsiri Dumrongpisutikul, Michael A Eller, Brandie Fullmer, Saowanit Getchalarat, Robert Gorelick, Barney Graham, Pornsuk Grandin, Surat Jongrakthaitae, Krisada Jongsakul, Shelly Krebs, Julie Ledgerwood, Sukalaya Lerdlum, Sopark Manasnayakorn, Corinne McCullough, Adrian McDermott, Mark Milazzo, Kayvon Modjarrad, Bessara Nuntapinit, Robert O'Connell, Kier Om, Madelaine Ouellette, Bijal Patel, Dominic Paquin-Proulx, Praphan Phanuphak, Nongluck Sangnoi, Alexandra Schuetz, Shida Shangguan, Mark de Souza, Sunee Sirivichayakul, Hiroshi Takata, Kamonkan Tangnaree, Rasmi Thomas, Andrey Tokarev, Nipattra Tragonlugsana, Randall Tressler, Rapee Trichavaroj, Sasiwimol Ubolyam, Phandee Wattanaboonyongcharoen, and Thipvadee Yamchuenpong.

Supplemental references

1. Doria-Rose NA, Altae-Tran HR, Roark RS, Schmidt SD, Sutton MS, Louder MK, et al. Mapping Polyclonal HIV-1 Antibody Responses via Next-Generation Neutralization Fingerprinting. *PLoS Pathog.* 2017;13(1):e1006148.
2. Yoon H, Macke J, West AP, Jr., Foley B, Bjorkman PJ, Korber B, et al. CATNAP: a tool to compile, analyze and tally neutralizing antibody panels. *Nucleic Acids Res.* 2015;43(W1):W213-9.
3. Rolland M, Edlefsen PT, Larsen BB, Tovanabutra S, Sanders-Buell E, Hertz T, et al. Increased HIV-1 vaccine efficacy against viruses with genetic signatures in Env V2. *Nature.* 2012;490(7420):417-20.
4. Katoh K, and Standley DM. MAFFT multiple sequence alignment software version 7: improvements in performance and usability. *Mol Biol Evol.* 2013;30(4):772-80.
5. Larsson A. AliView: a fast and lightweight alignment viewer and editor for large datasets. *Bioinformatics.* 2014;30(22):3276-8.
6. Price MN, Dehal PS, and Arkin AP. FastTree 2--approximately maximum-likelihood trees for large alignments. *PLoS One.* 2010;5(3):e9490.
7. Deng W, Maust BS, Nickle DC, Learn GH, Liu Y, Heath L, et al. DIVEIN: a web server to analyze phylogenies, sequence divergence, diversity, and informative sites. *Biotechniques.* 2010;48(5):405-8.
8. Robb ML, Eller LA, Kibuuka H, Rono K, Maganga L, Nitayaphan S, et al. Prospective Study of Acute HIV-1 Infection in Adults in East Africa and Thailand. *N Engl J Med.* 2016;374(22):2120-30.
9. Henikoff S, and Henikoff JG. Amino acid substitution matrices from protein blocks. *Proc Natl Acad Sci U S A.* 1992;89(22):10915-9.
10. Zhou T, Georgiev I, Wu X, Yang ZY, Dai K, Finzi A, et al. Structural basis for broad and potent neutralization of HIV-1 by antibody VRC01. *Science.* 2010;329(5993):811-7.
11. Zhou T, Zhu J, Wu X, Moquin S, Zhang B, Acharya P, et al. Multidonor analysis reveals structural elements, genetic determinants, and maturation pathway for HIV-1 neutralization by VRC01-class antibodies. *Immunity.* 2013;39(2):245-58.
12. Stewart-Jones GB, Soto C, Lemmin T, Chuang GY, Druz A, Kong R, et al. Trimeric HIV-1-Env Structures Define Glycan Shields from Clades A, B, and G. *Cell.* 2016;165(4):813-26.

13. Jones E, Oliphant E, Peterson P, and al. e. SciPy: Open Source Scientific Tools for Python. 2001.
14. Hamelryck T, and Manderick B. PDB file parser and structure class implemented in Python. *Bioinformatics.* 2003;19(17):2308-10.
15. Hunter JD. Matplotlib: A 2D graphics environment. *Computing In Science & Engineering.* 2007;9(3):90.
16. Cock PJ, Antao T, Chang JT, Chapman BA, Cox CJ, Dalke A, et al. Biopython: freely available Python tools for computational molecular biology and bioinformatics. *Bioinformatics.* 2009;25(11):1422-3.
17. Kluyver T, Ragan-Kelley B, Perez F, Granger B, Bussonier M, Frederic J, et al. Jupyter Notebooks – a publishing format for reproducible computational workflows. *Loizides, Fernando and Scmidt, Birgit (eds) In Positioning and Power in Academic Publishing: Players, Agents and Agendas IOS Press.* 2016.
18. Waskom M, Botvinnik O, O'Kane D, Hobson P, Ostblom J, Connell R, et al. mwaskom/seaborn: v0.9.0 2018.
19. Paradis E, and Schliep K. ape 5.0: an environment for modern phylogenetics and evolutionary analyses in R. *Bioinformatics.* 2019;35(3):526-8.
20. Wickham H. *Springer-Verlag New York.* 2009.
21. Grolemund G, and Wickham H. Dates and Times Made Easy with lubridate. *Journal of Statistical Software.* 2011;40(3):1-25.
22. Wickham H, François R, Henry L, and Müller K. dplyr: A Grammar of Data Manipulation. *R package version 083.* 2019.
